# The genetics of gout: translation into clinical practice

**DOI:** 10.1177/1759720X251366360

**Published:** 2025-08-25

**Authors:** Tony R. Merriman, Fiorella Rosas-Chavez, Lisa K. Stamp

**Affiliations:** Division of Clinical Immunology and Rheumatology, University of Alabama at Birmingham, Birmingham, AL, USA; Department of Microbiology and Immunology, University of Otago, Dunedin 9054, New Zealand; Division of Clinical Immunology and Rheumatology, University of Alabama at Birmingham, Birmingham, AL, USA; Department of Rheumatology, Immunology and Allergy, Te Whatu Ora, Waitaha, New Zealand; Department of Medicine, University of Otago Christchurch, Christchurch, New Zealand

**Keywords:** genetics, gout, Mendelian randomization, prediction, urate

## Abstract

Gout results from an innate immune response to monosodium urate crystals deposited in joints in people with hyperuricemia. Central to this is activation of the NLRP3 inflammasome and secretion of interleukin-1β. The pathogenic mechanism of NLRP3 inflammasome activation in gout is not well understood. However, recent genome-wide association studies (GWAS) in gout have revealed new pathogenic pathways, for example, genes involved in NLRP3 inflammasome activation and activity, and genes involved in clonal hematopoiesis of indeterminate potential. Genetic risk variants identified by GWAS are being used in Mendelian randomization studies to understand putative causal relationships between gout and co-morbid conditions (e.g., insulin resistance is causal of hyperuricemia). The genetic risk variants can also be combined into a genetic risk score to predict outcome in gout. Finally, inherited genetic variants influence response to allopurinol, in particular the p.Gln141Lys variant in ABCG2.

## Plain language summary

The genetics of gout: translation into clinical practice

Gout results from an innate immune response to monosodium urate crystals deposited in joints in people with hyperuricemia. Central to this is activation of the NLRP3 inflammasome and secretion of interleukin-1b. The pathogenic mechanism of NLRP3 inflammasome activation in gout is not well understood. However recent genome-wide association studies (GWAS) in gout have revealed new pathogenic pathways, for example genes involved in NLRP3 inflammasome activation and activity, and genes involved in clonal hematopoiesis of indeterminate potential. Genetic risk variants identified by GWAS are being used in Mendelian randomization studies to understand putative causal relationships between gout and co-morbid conditions (for example, insulin resistance is causal of hyperuricemia). The genetic risk variants can also be combined into a genetic risk score to predict outcome in gout. Finally, inherited genetic variants influence response to allopurinol, in particular the p.Gln141Lys variant in ABCG2.

## Introduction

Gout is a chronic disease of articular monosodium urate (MSU) deposition as a consequence of hyperuricemia. The classical clinical feature, the gout flare, is caused by an innate immune system reaction to the MSU crystals. Activation of the NLRP3 inflammasome and production of bioactive interleukin-1β is central to the gout flare. Gout can be compartmentalized into four stages^1^; hyperuricemia without MSU crystal deposition, asymptomatic MSU crystal deposition, MSU crystal deposition with gout flares, advanced gout (e.g., tophaceous disease). Directly related to the ease of assimilating very large cohorts of people with serum urate levels measured and with gout status adjudicated, the genetic basis of hyperuricemia and MSU crystal deposition with gout flares (i.e., the first and third stages) is becoming better understood. In contrast, the genetic basis of the second and fourth stages is unknown. Here, we will begin by broadly reviewing what is known about the genetic contribution to hyperuricemia and the gout flare. We will then focus on clinical applications of this knowledge, in particular the applications of the genetic epidemiological approaches Mendelian randomization and prediction using polygenic risk scores, and new drug targets identified by genome-wide association studies. Finally, we will review the genetic basis of response to the urate-lowering therapy allopurinol.

## The genetic basis of hyperuricemia and gout

A genome-wide association study (GWAS) uses millions of common genetic variants, typically single nucleotide polymorphisms (SNPs), spread throughout the human genome. The SNPs are simultaneously tested for association with a phenotype, for example, the linear outcome of serum urate levels or the binary outcome of gout. A GWAS is an unbiased approach based on the hypothesis that inherited genetic variants influence phenotype.

### Genetics of urate

For serum urate, the first GWAS were published in 2007–2008, identifying the major genetic effects at the *SLC2A9* and *ABCG2* loci that encode renal and gut uric acid transporters.^2,3^ Throughout, the term urate-raising allele refers to a specific DNA variant. For example, the urate-raising allele at *ABCG2* is the T-allele of the *rs2231142* SNP that encodes lysine at position 141 of the protein. Because this is the causal allele, it has the same effect in different populations. However, if the urate-associated SNP is not causal but is a marker for (i.e., in ‘linkage disequilibrium’ with) an unidentified causal variant the urate-increasing allele at such a SNP may be different between populations owing to differing linkage disequilibrium patterns.

In Europeans, each urate-raising allele of *SLC2A9* increases urate by 0.37 mg/dL (0.022 mmol/L) and the *ABCG2* urate-raising allele by 0.22 mg/dL (0.013 mmol/L).^4^ Predictably these alleles, and other urate-associated alleles detected by larger GWAS,^4^ associate with gout with a magnitude of risk for gout approximately proportionate to their effect on urate levels.^4,5^ More recent GWAS, that include people not of European ancestry, have collectively identified >300 loci associated with urate levels.^6,7^ Aside from loci harboring urate transporter genes, the role in urate homeostasis of the causal gene(s) at each of these loci remains unclear. An omnigenic model of polygenic control of urate has been proposed.^8^ In this model, core genes encode urate transporters that directly influence urate levels. The expression of the core genes is influenced by genetically associated transcription factors (e.g., *HNF4A*^7^ and *MAF*^9^) and interconnected gene regulatory networks that affect the function of the core genes.^8^

The majority (>80%) of genetic control of a complex phenotype is mediated by control of gene expression.^10^ Genetic control of gene expression ranges from epigenetic control of chromatin accessibility to binding and activity of transcription factors to control of RNA splicing. Common genetic variants (>0.1% minor allele frequency) that directly influence protein structure (e.g., missense variants) are likely causal at 10%–20% of disease loci.^11^ These genetic variants are the low-hanging fruit as, compared to variants acting via control of gene expression, they are straightforward to identify and directly indicate the causal gene/protein. In urate control and gout risk the p.Gln141Lys variant (rs2231142) in ABCG2 is a good example, with the lysine allele leading to reduced urate excretion.^12^ The AGCG2 141Lys allele, which associates with increased urate level and increased risk of gout, is causal of ~50% reduced secretion of urate.^13^ ABCG2 with the p.141Lys variant is fully processed but remains in the aggresome where mis-folded proteins aggregate prior to degradation^14^ ABCG2 secretes urate in the gut, with lysine allele-mediated dysfunction causing reduced gut excretion which, by overloading the renal uric acid excretion machinery, results in increased urinary uric acid excretion.^15,16^

In order to systematically identify missense variants, including rarer variants, whole exome sequence data can be scanned. This approach has revealed uncommon variants with strong effect on urate levels in *XDH* (the gene encoding the urate producing enzyme xanthine oxidoreductase) and the urate transporter genes *SLC2A9* (encodes GLUT9), *SLC22A12* (encodes URAT1) and *ABCG2*.^17^ All variants were predicted to cause reduced or loss of function of the respective genes, with the variants in *XDH, SLC2A9*, and *SLC22A12* associated with reduced urate levels and those in *ABCG2* associated with increased urate levels. This is consistent with the function of the respective proteins—reduced xanthine oxidoreductase activity would result in less urate production, reduced GLUT9 and URAT1 activity would result in less urate being reabsorbed from filtered urine, and reduced ABCG2 activity would result in less excretion of urate in the gut and kidney.

### Genetics of gout

**Figure 1.**
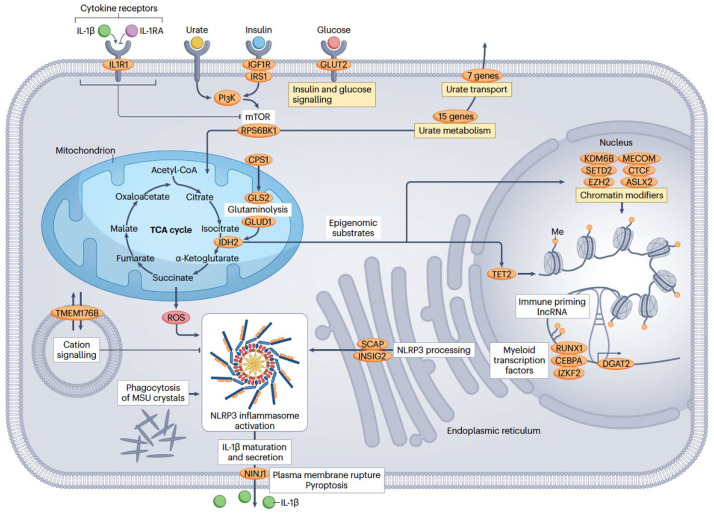
New pathogenic pathways identified in gout. Source: Taken with permission from Leask et al.^19^

GWAS of gout have lagged behind those of serum urate. The largest GWAS in gout to date included 120,295 people with gout and detected 377 loci (29 only in men and 8 only in women) and revealed new candidate pathogenic pathways^11^ (Figure 1). Two previous large GWAS have been reported in gout, one with 13,170 cases^7^ and the other with 37,105 cases.^18^ The former study had a focus on genetic control of urate level and did not discover any loci implicated in gouty inflammation. The latter study, that included gout among 14 phenotypes, reported 52 gout-associated loci, 40 of which had previously been associated with urate. The Zhou et al. study^18^ did not further characterize any of the gout-associated loci.

In the Major et al. GWAS,^11^ there were no loci harboring genes encoding constituents of the NLRP3 inflammasome; however, there were genes identified with roles in regulating the formation and activity of the inflammasome and roles in pyroptosis (programmed cell death in inflammation). Examples are *TMEM176B* (encoding a transmembrane calcium channel that inhibits NLRP3 inflammasome activation), *SCAP* (the SCAP–SREBP2 complex activates the inflammasome), *INSIG2* (encodes a binding partner of SCAP), *NINJ1* (its product regulates the NLPR3 inflammasome during the MSU crystal response), *GNAI3* (encoding inhibitory G protein subunit), *TNN* (encoding tenascin-C), SPRY1 (encoding sprouty RTK signaling antagonist 1) and *CDNK1A* (encoding cyclin-dependent kinase inhibitor 1A). There were a number of genes, for example *TET2* and *ASXL2*, known to harbor somatic mutations that are risk factors for clonal hematopoiesis (of myeloid cells), a risk factor for hematological malignancy. Clonal hematopoiesis has been associated with gout.^20^ These genes, and others identified in the GWAS, are involved in epigenomic remodeling of the genome (e.g., methylation/demethylation, histone modification). Epigenomic remodeling of monocytes has been implicated in enhancing immune response to MSU crystals.^21^

The gout GWAS reported 22 loci associated with gout but not urate, consistent with a role in the inflammatory process of gout.^11^ Notable among these loci included those harboring the *IL1R1, IL1RN, CSF1*, and *IL6R* genes. Intriguingly, a further 65 loci were associated with urate, but despite sufficient power to detect association with gout, were not associated with gout at a *p* < 0.01 level of significance.^11^ Biologically, this is perplexing given the incontrovertible relationship between increased urate level and increased risk of gout. This may reflect a phenomenon where the urate-raising alleles have pleiotropic effects, lowering the risk of gout through a separate inflammatory pathway. Two of these loci include *IL33* and *IL1R1*—interleukin-33 ameliorates MSU crystal-induced inflammation^22^ and signals through IL-1R1. The urate-increasing alleles at each locus associate with increased expression of each gene, which can be predicted to reduce risk of gouty inflammation. One other locus included *RCOR1*, a regulator of the REST transcription factor and a mediator of gene regulation and chromatin remodeling. This gene had previously been identified in a genome-wide association study of adolescent-onset gout in China.^23^ The expression of *RCOR1* in monocytes is required for induction of IL-1β after exposure to MSU crystals,^23^ and there are REST transcription factor binding sites in the promoter/enhancer regions of the gout-associated *CSF1* and *CSF1R* loci (*CSF1* encodes colony-stimulating factor-1, required for the differentiation of macrophages from monocytes).

Based on physiologic drivers of hyperuricemia gout can be clinically stratified into four subtypes^15^; renal underexcretion, renal overload (urate over-production and/or reduced gut excretion), and ‘normal’. A Japanese gout cohort of 3053 cases was stratified into the four subtypes and a separate GWAS conducted for each subtype.^24^ The only locus detected at a genome-wide level of significance in all four groups was *ABCG2*. The effect was more pronounced in the renal overload group, consistent with the primary role of ABCG2 in gut excretion of urate. Two novel loci with a very strong effect (OR > 4) were detected in the ‘normal’ group. The loci contain genes encoding immune-related surface antigens and candidate urate transporters. This strategy of clinical subtyping of gout prior to GWAS has considerable merit and could generate further insights in larger studies.

## Clinical translation: New drug targets for gout inflammation from recent GWAS, some examples

A notable signal from the Major et al. GWAS in gout^11^ was at the receptor for IL-6 (IL6R). This signal was present in gout but not associated with urate level suggesting a role for the IL-6R in the inflammatory process of gout. IL-6R is targeted by the immunosuppressive monoclonal antibody tocilizumab, used in rheumatoid arthritis and juvenile idiopathic arthritis. Several case reports indicate efficacy of tocilizumab in refractory gout.^25–27^ With the caveat that case reports are inherently limited by very small sample and potential publication bias, this suggests that tocilizumab could be more widely evaluated in the management of refractory gout. Interestingly, IL-6R is also implicated in atherosclerosis mediated by clonal hematopoiesis. Blockade of IL-6R and IL-6 signaling reduces atherosclerosis in mice when *Tet2* is deficient by reducing expression of *Csf1r*.^28^ Understanding the molecular mechanism(s) of the role of IL-6R in gout, perhaps mediated by IL-6 signaling and/or a role in clonal hematopoiesis, will be important in evaluating the suitability of tocilizumab as a therapy in gout.

Clonal hematopoiesis was causally implicated in gout using the Mendelian randomization technique (refer below) by Major et al.^11^ and has been associated with gout.^19^ Clonal hematopoiesis is also a risk factor for phenotypes including atherosclerosis and cardiometabolic disease and is associated with increased mortality.^29^ Drugs targeting proteins mutated in clonal hematopoiesis^30^ could be evaluated in gout.

Pyroptosis is a downstream phenomenon of NLRP3 inflammasome activation. It results from osmotic swelling and plasma membrane rupture that releases damage-associated molecular patterns (DAMPs) such as lactate dehydrogenase. The NINJ1 gene is associated with gout with the risk allele of lead SNP rs10761194 associated with increased expression of NINJ1.^11^ The NINJ1 gene encodes a protein integral to the formation of pyroptotic pores large enough to secrete DAMPs such as lactate dehydrogenase. In other phenotypes either genetic ablation or antibody inhibition of NINJ1 attenuates damage.^31^ NINJ1 is another of a number of new potential targets to manage gout.

## Clinical translation: Applications of genetic epidemiology

Not only do GWAS studies uncover new candidate causal genes pathways that can be evaluated for new therapeutic approaches, the phenotype-associated genetic variants can also be applied to clinical questions. Here, we will review two genetic epidemiological applications: (1) Mendelian randomization to test for causal relationships between hyperuricemia and gout and other co-morbid conditions, and (2) use of genetic risk scores in prediction of gout and outcome.

### Mendelian randomization

Hyperuricemia and gout are often co-morbid with cardiorenal conditions and their risk factors. There are many observational epidemiological studies reporting this association.^32^ This has led, in the absence of supporting clinical trial evidence for a proposed causal role of increased urate levels in the various co-morbidities, to suggestions that asymptomatic hyperuricemia should be regarded as a disease state and treated with urate-lowering therapy,^33^ for example, in Japan.^34^ Even in the best designed observational studies identification of causal factors (unless they have very strong effect sizes) is not possible owing to confounding by unmeasured biological factors and environmental exposures. To overcome this limitation in epidemiological approaches, nonconfounded surrogates are required for exposures (i.e., hyperuricemia and gout), which can be provided by exposure-associated genetic variants randomly inherited at conception and not influenced by confounders. The random inheritance, as explained by the second law of independent assortment of genes by Mendel, is analogous to the random assignment of participants to different exposures in a randomized controlled trial. In application to hyperuricemia and gout, the “intervention group” would be those who inherit a urate-increasing or gout risk allele, and the “control group” would be those who inherit the other allele. These risk alleles, or “instrumental variables,” can be tested for causal association with exposure using the technique of Mendelian randomization. The fundamental basis is that, if an exposure is causal of an outcome, then a genetic variant associated with the exposure should also be associated with the outcome.

Mendelian randomization rose to prominence in biomedical research in the 2010s, for example, to provide evidence against a causal relationship between high-density lipoprotein cholesterol and myocardial infarcation,^35^ and to provide evidence supporting a causal role for low-density lipoprotein cholesterol in coronary heart disease.^36^ The instrumental variable is commonly an aggregate of exposure-associated genetic variants identified by GWAS, and needs to satisfy several assumptions, the most important of which is that any causally associated instrumental variable must influence the outcome solely through the exposure. This assumption can be difficult to test and can be best mitigated by careful selection of the genetic variants included in the instrumental variable. For example, when constructing an instrumental variable for hyperuricemia one could include genetic variants from the urate transporter *SLC2A9, SLC17A1*, and *SLC22A12* loci for which there is little to no evidence for a role in other disease-related biological processes outside of uric acid transport. However, it would be inadvisable to include a genetic variant from the *ABCG2* locus given that there is evidence for a role of ABCG2 in disease-related biological processes outside of hyperuricemia.^12^ Initially, Mendelian randomization studies used linear or logistic regression or, if individual level data were available, two-stage least squares would be used where the genetic variant is regressed against the exposure in stage 1 and fitted values from stage 1 regressed against the outcome of interest in stage 2. The two-stage least squares method has the advantage of giving an estimate of the effect size of the exposure on the outcome. More recently, however, the dominant approach has been two-sample Mendelian randomization that takes advantage of readily accessible GWAS summary statistics from, for example, the UK Biobank,^37^ FinnGen,^38^ and the Million Veterans Program.^39^ Application of two-sample Mendelian randomization has been commoditized by the availability of user-friendly statistical tools, for example, MendelianRandomization in R.^40^ The easy access to datasets and analytical tools has led to a recent explosion of studies applying Mendelian randomization to causal questions in urate/gout with the recent literature essentially uninterpretable for nonexperts.^41^ Stender et al.,^42^ in a article entitled “Reclaiming mendelian randomization from the deluge of papers and misleading findings,” state that “These studies add minimal—if any—value and overwhelm reviewers and journals.” Stender et al. advise editors to reject without review two-sample MR papers that only report the MR findings per se with no additional supporting evidence. We fully support these views. We have developed a metric for assessing quality of Mendelian randomization studies in urate and gout, which can be applied to the wider Mendelian randomization literature.^41^

Here, we summarize in Table 1 selected Mendelian randomization studies in urate and gout. Some are high-quality recent two-sample Mendelian randomization studies previously identified by us^41^ and others are selected largely from the earlier Mendelian randomization literature. There are more associations shown for urate than gout, reflecting the current literature. This is because summary statistics of large GWAS for serum urate have been available for longer, whereas large GWAS for gout are only more recent. Mendelian randomization studies have robustly identified body mass index as a causal risk factor of increased urate concentrations, with this relationship possibly mediated by the effect of insulin resistance on urate transport.^43^ Both metformin use and physical activity causally associate with reduced urate levels. There is increasing evidence that urate causally associates with increased risk of heart disease, but not chronic kidney disease. The latter findings are consistent with randomized clinical trials that demonstrated no effect of urate-lowering drugs on progression of chronic kidney disease.^44–46^ There is no evidence by Mendelian randomization that urate causally associates with type 2 diabetes; however, there is evidence that increased fasting insulin associates with hyperuricemia and increased risk of gout.^47^

**Table 1.** Robust Mendelian randomization studies in hyperuricemia and gout.

Ancestry	Exposure	Outcome	Strength of association, if any	PMID
Risk factors for hyperuricemia or gout				
European	BMI	Serum urate Gout	0.30 (0.25 to 0.35) mg/dL increase in serum urate (SU), OR 2.24 (1.70 to 2.95) increase in gout risk per 4.6 kg/m^2^ increase in BMI	30085130^48^
East Asian	BMI	Serum urate	β^a^ = 0.18 (0.11 to 0.24)	36709979^49^
European	Fasting insulin	Serum urate Gout	0.37 mg/dL per log unit increase in fasting insulin OR 1.07 (1.05 to 1.09) for gout per standard deviation (SD) increase in fasting insulin genetic score	33982892^47^
European	Metformin use	Serum urate Incident gout	β = −0.25 (−21.4 to 4.2) µmol/L per 0.62% decrease in HbA1c No association with gout	37807832^50^
European	Physical activity (measured by accelerometer; m/s^2^)	Serum urate Gout	β = −0.339 (−0.522 to −0.156) mg/dL per 1 − SD increment in genetic risk score No association with gout	35622233^51^
European	Systolic blood pressure Diastolic blood pressure	Gout	OR 1.02 (1.01 to 1.03) No association	38841306^52^
Conditions for which hyperuricemia is a risk factor				
European	Serum urate	Heart failure	OR 1.07 (1.03 to 1.1) per 1 mg/dL increase in genetically predicted SU	35578763^53^
European	Serum urate	Coronary heart disease Peripheral artery disease Stroke	OR 1.19 (1.10 to 1.30) OR 1.12 (1.03 to 1.21) OR 1.11 (1.5 to 1.18) per SD increase in genetically predicted SU	33356394^54^
European	Serum urate	Coronary heart disease	No association	25634581^55^
European	Serum urate	Triglycerides	No association	25249548^56^
European	Serum urate	Fasting insulin	No association	33982892^47^
European	Serum urate	Type 2 diabetes mellitus	No association	25918230^57^
European	Serum urate	Type 2 diabetes mellitus	No association	21717115^58^
East Asian	Serum urate	Prostate cancer	OR 1.18 (1.03 to 1.36) per 1.4 mg/dL increase in SU	36542132^59^
European	Serum urate	Chronic obstructive pulmonary disease	OR 1.15 (1.03 to 1.11)	3847981^60^
European	Serum urate	Chronic kidney disease	No association	30645594^61^
European	Serum urate	Renal function (estimated glomurelar filtration rate)	β = 12.2 µmol/L Cr per mmol/L increase in SU	24048376^62^

a A unitless ratio of the genetic association with the exposure and the genetic association with the outcome, derived from two-sample Mendelian randomization.

### Prediction of gout using a genetic risk score

Since the advent of GWAS, there has been much interest in the potential utility of genetic (polygenic) risk scores to predict onset and outcomes of complex disease. A genetic risk score (GRS) is based on a defined set of inherited genetic variants associated with a disease. A GRS is typically identified by GWAS and is a summation over all genetic variants included of the number of risk alleles at each locus (zero, one, or two) multiplied (i.e., weighted) by their effect on disease (i.e., odds ratio). The GRS can then be included in predictive models. In gout, the first use of this was by Tin et al.^7^ In a European-ancestry dataset, they used 183 urate-associated genetic variants to predict prevalent gout—this gave an area under the receiver operating curve (AUROC) of 0.67, far too low to be clinically useful. When added to a demographic model of age and sex the GRS increased the AUROC from 0.80 to 0.84. Very similar data were obtained using gout-associated variants from a gout GWAS.^11^ Thus, for predicting prevalent gout a GRS may only be useful when added to existing clinical models. A practical scenario may be in a primary care environment with access to genome-wide genotype information of patients. A GRS, based on 19 gout-associated SNPs identified from a GWAS in the UK Biobank,^63^ has been associated with the presence of tophi in men with gout in European and Polynesian (Aotearoa New Zealand Māori and Pacific peoples) people, but not women.^63^ However, the GRS has not yet been evaluated (with and without other clinical variables) for its utility in predicting the development of tophus in people with gout. A gout-specific GRS (i.e., genetic variants associated with gout but not hyperuricemia) could be part of a model to predict onset of gout in people with hyperuricemia. Once GWAS are done for outcomes in gout (e.g., flare frequency and development of tophus) a consequent outcome-associated GRS could be tested for utility to predict outcomes in newly diagnosed people with gout.

### Prediction of allopurinol response using genetic variants

Genome-wide genetic approaches have been used to detect genetic variants associated with response to the widely used urate-lowering drug allopurinol. Allopurinol, which inhibits the enzyme xanthine oxidoreductase that converts xanthine to urate is the first-line therapy for gout. The American College of Rheumatology gout management guidelines recommend a target serum urate level of less than 0.36 mmol/L (6 mg/dL) to dissolve MSU crystals and prevent gout flares.^64^ Many people with gout fail to achieve target serum urate on allopurinol.^65^ The most common underlying reasons are poor adherence and persistence with therapy and under dosing. However, there is a small group of people who fail to achieve target serum urate even when allopurinol is appropriately dose escalated, and they are adherent with therapy.^65^

Failure to achieve target urate with allopurinol could be influenced by inherited genetic variants. To investigate this possibility, a GWAS first identified the p.Gln141Lys variant (encoded by rs2231142) of the uric acid transporter ABCG2 to associate with poorer response to allopurinol, defined as change from baseline serum urate using administrative data.^66^ This finding was corroborated in a second GWAS^67^ and replicated in datasets where adherence to allopurinol was confirmed.^68,69^ Confirmation was established by measuring the active metabolite (oxypurinol) in the serum and where a good response was defined as both the achieving of target serum urate <6 mg/dL with a daily dose of allopurinol ⩽300 mg.^68,69^ The association of the ABCG2 p.Gln141Lys allele with partial allopurinol response was independent of BMI, kidney function (eGFR), and baseline urate.^68,69^ Consistent with these findings a Canadian study reported association of the ABCG2 p.Gln141Lys allele with increased allopurinol dose in people with gout.^70^ A Chinese study reported no association of the ABCG2 p.Gln141Lys variant with allopurinol response.^71^ However, although the definition of response was the composite of target urate and allopurinol dose, there was no measure of adherence, and it appears they included people who had poor compliance with allopurinol, an effect that is likely to bias the results.

Participants in a prospective clinical study, homozygous for the ABCG2 p.Gln141Lys allele exhibited a longer half-life of oxypurinol^72^ which, at face value, conflicts with the genetic association data. A longer half-life of oxypurinol resulting from the ABCG2 p.Gln141Lys allele would be expected to increase the efficacy of allopurinol. Finally, there was no evidence for association between the ABCG2 p.Gln141Lys allele and serum oxypurinol levels and clearance (pharmacokinetics) in clinical studies using multivariable-adjusted models (although there was evidence for association with lower levels and increased clearance in a univariable model).^73,74^

Collectively, there is strong evidence that the ABGC2 p.Gln141Lys allele is associated with poorer response to allopurinol. Of potential clinical utility, inclusion of ABCG2 rs2231142 genotype in an allopurinol dosing tool could improve performance of the tool.^75^ The only other genetic association reported with partial allopurinol response is with rare and common variants in the MOCOS gene.^76^ MOCOS encodes MOCO sulfurase which sulfates molybdenum cofactor (MOCO), required for xanthine oxidoreductase and aldehyde oxidase activity. This could affect the conversion of allopurinol to oxypurinol.

To date, the mechanism of the effect of ABCG2 p.Gln141Lys on allopurinol response is not understood. ABCG2 is an efflux pump for both allopurinol and oxypurinol with preferential transport of oxypurinol compared to allopurinol caused by the ABCG2 glutamine allele in HEK293 cells.^66^ In the same experimental system of HEK293 cells, in those containing the ABCG2 lysine variant, there was also impaired transport; however, the effects were equal between allopurinol and oxypurinol.^66^ Nakamura et al.^77^ reported that ABCG2 transports oxypurinol but not allopurinol (this is not entirely inconsistent with the results of Wen et al.,^66^ using the common glutamine variant of ABCG2). Wen et al. suggested that the lysine allele could increase the concentration of allopurinol and oxypurinol in renal tubule cells and decrease the concentration in filtered urine, thus reducing the inhibitory effect of uric acid on renal reuptake transporters.^66^ It is important to acknowledge that data from cell lines cannot accurately model complex genetic pharmacokinetics of oxypurinol.

Most importantly for allopurinol, the presence of HLA*5801 has been consistently associated with the feared and potentially fatal adverse reaction known as allopurinol hypersensitivity syndrome (AHS).^78^ AHS, which is characterized by rash, eosinophilia, leukocytosis, fever, hepatitis, and progressive kidney failure^79^ is rare, occurring in ~0.1% of people, but has a high morbidity and mortality. A number of risk factors have been identified including impaired kidney function, starting dose of allopurinol, concomitant diuretic therapy, and the presence of HLA-B*5801.^80^ The prevalence of HLA-B*5801 is particularly high in people of Han Chinese, Korean, and Thai descent and HLA-B*5801 screening in these high-risk populations is recommended; if positive, allopurinol should be avoided and another urate-lowering therapy selected.^81^

## Conclusion

Recent GWAS in serum urate and gout have revealed new pathogenic pathways. Gout- and urate-associated genetic variants identified by GWAS can be applied to epidemiological questions—can the risk of gout be predicted, and to the question of causal relationships between urate and gout and co-morbid conditions. Finally, genetics has yielded insights into the genetic basis of allopurinol resistance.

## Declarations

**Ethics approval and consent to participate:** Not applicable.

**Consent for publication:** Not applicable.
